# Gene-replacement therapy in neurodevelopmental disorders: progress and challenges

**DOI:** 10.1172/JCI188703

**Published:** 2025-02-03

**Authors:** Holger Lerche, Ulrike B.S. Hedrich, Thomas V. Wuttke

**Affiliations:** 1 Department of Neurology and Epileptology, Hertie Institute for Clinical Brain Research, and; 2 Department of Neurosurgery, University of Tübingen, Tübingen, Germany.

## Abstract

Heterozygous loss-of-function variants in the *SLC6A1* gene, encoding GAT1, which is the main GABA transporter in the brain, lead to a broad spectrum of neuropsychiatric and neurodevelopmental disorders including epilepsy, developmental delay, intellectual disability, and autism. Gene-replacement strategies involving adeno-associated viruses (AAV) require the delivery of genes to specific types of neurons or areas in the brain, likely during certain developmental time points. In this issue of the *JCI,* Guo and colleagues from the Gray lab evaluated two promoters, three injection modalities, and various timing strategies for replacement of GAT1 via AAV type 9 in heterozygous and homozygous knockout mouse models. Intrathecal administration of vectors containing either promoter at postnatal day 5 achieved high expression and was the best tolerated approach. Notably, gene-replacement therapy failed at later disease stages, suggesting the importance of early gene reconstitution and confirming the importance of GABA metabolism in early brain development.

## Optimizing gene replacement

Disease caused by complete or partial loss of function of the protein encoded by the affected gene may, conceptually, be treated by the replacement of the missing gene. This gene-replacement strategy has been clinically successful, leading to the first licensed virally mediated gene replacement for a severe neurological disease, spinal muscular atrophy type 1 (SMA1), which is caused by loss of *SMN1* in spinal motoneurons (1). *SMN1* can be successfully replaced as soon as possible after diagnosis via i.v. application of an adeno-associated virus type 9 (AAV9), reaching a sufficient amount of SMN1 in spinal motoneurons of neonates or infants. Meanwhile, a couple of other virally mediated gene-replacement therapies in severe neurological disorders have been licensed or are still under clinical development (2). However, the challenges that we have to overcome to render such therapies successful are numerous, including crossing of the blood-brain barrier (BBB) for i.v. applications, delivery to the critical brain areas and the affected types of neurons, achieving therapeutic gene expression dosage, and – particularly in neurodevelopmental disorders – treating during an appropriate developmental time window.

**Figure 1:**
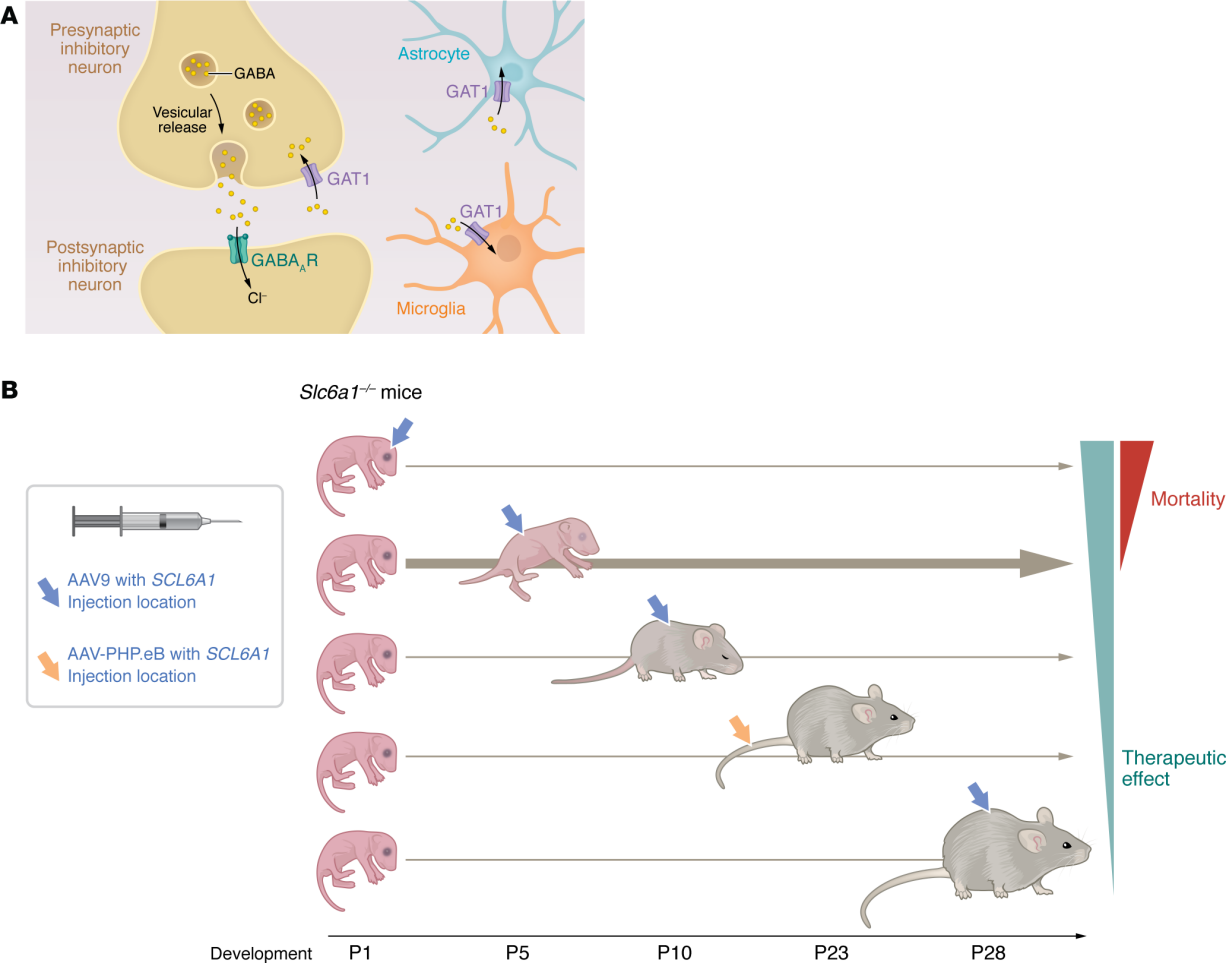
GAT1 is responsible for GABA reuptake after release from presynaptic nerve terminals and was replaced at different developmental time points. (**A**) In the presynaptic neuron, vesicles are packed with GABA and GABA is released into the synaptic cleft upon membrane depolarization and activation of the presynaptic release machinery. At the postsynaptic membrane, it binds to and activates ionotropic GABA_A_ receptors mediating synaptic inhibition. Released GABA is cleared by the GABA transporter GAT1, which is primarily expressed in neurons, but also in glial cells (astrocytes and microglia). (**B**) Evaluation of GAT1 restoration to Slc6a1 knockout mice using AAV vector-based gene therapy involved different injection sites and developmental time points (ICV at P1, intrathecal at P5, P10, and P28, and intravenously [tail vein] at P23). Intrathecal injection at P5 was adopted to balance the risk for mortality, which occurred at earlier time points with the therapeutic effect that diminished with development (3).

In this issue of the *JCI*, Guo et al. report about exploring the aforementioned difficulties for a virally mediated replacement of GAT1 (3), the main GABA transporter of the brain. GAT1, encoded by the gene *SLC6A1*, mediates reuptake of GABA after synaptic release in the presynaptic inhibitory nerve terminals (Figure 1A). Heterozygous loss-of-function variants in this gene lead to a broad spectrum of neuropsychiatric and neurodevelopmental disorders, including severe epilepsy, developmental delay, intellectual disability, and autism. Functional assays of all known disease-associated variants using uptake assays and surface expression excluded gain-of-function as a relevant disease mechanism (4). To replace the loss-of-function gene product, Guo et al. (3) used an AAV9 for delivery of *SLC6A1* into mouse models. The Gray lab has done pioneering work using AAV9 (and other vectors) and optimizing gene delivery to the nervous system. Through preclinical studies and phase 1 trials, the group drives the advancement of such technologies into therapeutic solutions for neurological diseases, focusing on global, efficient, and, in some cases, cell-type–specific CNS gene delivery (5, 6).

Guo et al. (3) first searched for stable experimental conditions by examining the phenotypes for various mouse models, including heterozygous and homozygous *Slc6a1* KO and 2 different missense variants. The complete *Slc6a1* KO and missense variants showed very similar phenotypes with frequent epileptiform discharges on EEG recordings, behavioral seizures, and a variety of abnormal behaviors, including nest building, open field exploration, fear conditioning and motor tasks. Homozygous animals were consistently more affected than heterozygous ones, with heterozygous animals not representing the full spectrum of human disease. The authors finally chose the herozygous and homozygous *Slc6a1* KO models for their study, since overall those showed the most consistent and reproducible phenotypes (3).

Guo and colleagues investigated several routes of delivery and developmental time points for viral gene transfer (3) (Figure 1B). They first tested intra-cerebro-ventricular (ICV) application of two alternative AAV9 vectors driving *SLC6A1* expression either under a ubiquitously acting synthetic weak promoter called JeT, or the predominant neuronal promoter MeP229 at P1. Both strategies were highly successful in almost completely diminishing seizure and EEG activity and restoring behavioral abnormalities in heterozygous and homozygous *Slc6a1* KO mice. However, half of the treated animals died for unknown reasons under the JeT promoter, and 17% under the MeP promoter. The authors therefore switched to an intrathecal (IT) application, and since a neonatal injection is unrealistic for a clinical application, they adopted injection at the P5 time point. This type of application (and possibly also the later injection) resolved the increased mortality but was less successful. While abnormal EEG, seizures, and behavioral parameters were largely improved for heterozygous animals, these abnormalities remained in homozygous animals (except for nest building). Similarly, when homozygous animals were treated later by IT application at P10 or P28, a therapeutic effect could not be observed. Finally, the authors tested i.v. application at P23 of a different AAV capsid, which better passes the BBB (named AAV-PHP.eB) and results in high expression of the target gene in the brain. Nevertheless, neither heterozygous nor homozygous animals benefited from this therapy (3).

## The importance of timing

Although the positive results for early treatment look promising and raise hope for translation, the question arises as to why gene-replacement therapy failed at later disease stages. Guo and colleagues controlled for gene expression in individual cells using RNAscope and single-cell RNA Seq (3). These analyses showed that *SLC6A1* was well expressed in central neurons at levels approximately corresponding to or even exceeding expression of the endogenous mouse gene in control animals in all the different conditions tested. In other words, a delivery problem could be largely excluded (although only 10%–20% of the affected neurons were transduced). It rather seems that the time point of gene reconstitution came too late and that proper neurodevelopment requires the correct and full function of GABA transporters early on; otherwise the neuronal and network architecture seems to be disturbed in an irreversible way. Other potential problems may arise from the ectopic expression of GABA transporters induced by the relatively broad promoters. GAT1 expression is usually restricted to inhibitory interneurons and some glial cells (7, 8), so that overexpression in all neurons or even more cell types might be counterproductive. On the other hand, the results with the ubiquitous JeT promoter were even better at P1 at least for homozygous animals than with the more neuron-specific MeP promoter, which argues a bit against the theory of a potentially harmful ectopic overexpression. So despite thoroughly testing different model systems, developmental time points, and promoters, further studies will be necessary to understand and solve these problems.

## Conclusions

In another disease with a different genetic defect, the reversibility of symptoms and parameters of epileptogenesis by a complete gene replacement has been shown recently. The research group of G. Colasante and V. Broccoli showed symptoms of Dravet syndrome were partially reversable in a conditional mouse model in which the KO of the Na^+^ channel encoding gene *Scn1a* could be switched off at different time points (9). Notably, symptoms and features of epileptogenesis were partially reversible until adult age of P90 (9). Since this example represents an optimal gene-replacement approach in the right cells with the endogenous promoter, thus providing physiological levels of gene dosage, these conditions may not translate to a gene therapy in humans. On the other hand, another study showed that the replacement of the same gene in the low amount of 6% of the affected interneurons via expression using an adeno-virus could also partially rescue phenotypes (10). There is also evidence that specific developmental time windows of opportunity exist to potentially cure neurodevelopmental disorders by restricting treatment to a narrow but functionally important time window, as has been shown for a K^+^ channel defect (although not using a gene therapy) (11). These studies reveal that we still have to understand the highly complex mechanisms of inducing a disease by gene deletion and a potential treatment, which probably depend on many factors including the developmental role of the gene of choice, its potential compensation by the regulation of other genes, and the reaction of the network. Gray’s group here contributes another important piece of that puzzle, confirming that GABA metabolism is highly important in early brain development (12), and that its disruption likely leads to irreversible damage within few weeks after birth.

## Version 1 02/03/2025

Electronic publication
